# An AICD-based functional screen to identify APP metabolism regulators

**DOI:** 10.1186/1750-1326-2-15

**Published:** 2007-08-24

**Authors:** Can Zhang, Preeti J Khandelwal, Ranjita Chakraborty, Trinna L Cuellar, Srikant Sarangi, Shyam A Patel, Christopher P Cosentino, Michael O'Connor, Jeremy C Lee, Rudolph E Tanzi, Aleister J Saunders

**Affiliations:** 1 Department of Bioscience & Biotechnology, Drexel University, Philadelphia, PA, USA; 2 Genetics and Aging Research Unit, MassGeneral Institute for Neurodegenerative Diseases (MIND), Department of Neurology, Massachusetts General Hospital, Harvard Medical School, Charlestown, MA, USA; 3 Department of Biochemistry & Molecular Biology, Drexel University College of Medicine, Philadelphia, PA, USA

## Abstract

**Background:**

A central event in Alzheimer's disease (AD) is the regulated intramembraneous proteolysis of the β-amyloid precursor protein (APP), to generate the β-amyloid (Aβ) peptide and the APP intracellular domain (AICD). Aβ is the major component of amyloid plaques and AICD displays transcriptional activation properties. We have taken advantage of AICD transactivation properties to develop a genetic screen to identify regulators of APP metabolism. This screen relies on an APP-Gal4 fusion protein, which upon normal proteolysis, produces AICD-Gal4. Production of AICD-Gal4 induces Gal4-UAS driven luciferase expression. Therefore, when regulators of APP metabolism are modulated, luciferase expression is altered.

**Results:**

To validate this experimental approach we modulated α-, β-, and γ-secretase levels and activities. Changes in AICD-Gal4 levels as measured by Western blot analysis were strongly and significantly correlated to the observed changes in AICD-Gal4 mediated luciferase activity. To determine if a known regulator of APP trafficking/maturation and Presenilin1 endoproteolysis could be detected using the AICD-Gal4 mediated luciferase assay, we knocked-down Ubiquilin 1 and observed decreased luciferase activity. We confirmed that Ubiquilin 1 modulated AICD-Gal4 levels by Western blot analysis and also observed that Ubiquilin 1 modulated total APP levels, the ratio of mature to immature APP, as well as PS1 endoproteolysis.

**Conclusion:**

Taken together, we have shown that this screen can identify known APP metabolism regulators that control proteolysis, intracellular trafficking, maturation and levels of APP and its proteolytic products. We demonstrate for the first time that Ubiquilin 1 regulates APP metabolism in the human neuroblastoma cell line, SH-SY5Y.

## Background

Alzheimer's disease (AD) is characterized by significant accumulation of cerebral amyloid plaques and intraneuronal neurofibrillary tangles. Amyloid plaques are composed mainly of the β-amyloid peptide (Aβ). Aβ is a normal product of amyloid precursor protein (APP) metabolism. Several genes have been identified encoding enzymes that directly metabolize APP to generate Aβ; however, it is not fully understood how APP metabolism is regulated. Here we describe and validate a novel experimental approach for identifying genes encoding regulators of APP metabolism.

Aβ is generated by the successive proteolytic processing of APP, a process referred to as regulated intramembrane proteolysis (RIP) [1-3]. RIP occurs when a transmembrane protein is cleaved within the transmembrane domain, releasing a cytoplasmic fragment that can activate gene expression in the nucleus [1]. RIP requires two cleavage events; the first, outside the membrane, often in response to ligand binding, can trigger the second, intramembraneous, cleavage. RIP liberates small, intracellular protein domains that are involved in nuclear signaling processes [1,2]. Therefore, regulation of RIP is critical for controlling nuclear signaling. Identifying the regulatory mechanisms controlling these proteolytic steps is important for a fuller understanding of these processes.

APP is a type I transmembrane glycoprotein and is suggested to function in neuroprotection, synaptic transmission, signal transduction, and axonal transport [4,5]. Upon being synthesized, APP undergoes maturation in the protein secretory pathway. APP is N-glycosylated in the ER and cis-Golgi followed by O-glycosylation in medial- and trans-Golgi. RIP of APP can occur via two alternative routes: amyloidogenic and non-amyloidogenic. In amyloidogenic processing, APP undergoes sequential cleavage by β-secretase (BACE) and γ-secretase to generate Aβ [6]. BACE cleavage occurs in the APP extracellular domain to produce a soluble extracellular fragment called sAPPβ and a membrane associated, 99-residue C-terminal fragment called C99 [7] The C99 fragment is a substrate for subsequent cleavage by the γ-secretase complex [8,9]. The active γ-secretase complex is composed of the amino- and carboxy-terminal fragments of presenilin1 (PS1), a highly glycosylated form of nicastrin (NCSTN), Aph1α and Pen-2 [8,9]. The amino- and carboxy-terminal fragments of PS1 (~27 and ~17 kDa respectively) are derived by endoproteolytic cleavage of the inactive, full length PS1 protein within the large hydrophilic loop that spans between transmembrane helices 6 and 7 and are thought to interact with each other [10]. The products of γ-secretase cleavage are the cytoplasmic APP Intracellular Domain (AICD) fragment and Aβ peptides of varying length, mainly 40 and 42 residues long [11-13]. In non-amyloidogenic processing, the initial extracellular cleavage of APP is catalyzed by one of a group of proteases termed α-secretases. These enzymes include ADAM9, ADAM10, and ADAM17 (TACE). α-secretase cleavage produces a soluble extracellular fragment called sAPPα and a membrane associated, 83-residue C-terminal fragment called C83. This C83 fragment is then cleaved by the γ-secretase complex to produce AICD and a p3 peptide, which is not involved in amyloidogenesis [6].

A common feature of RIP processing is the liberation of an intracellular protein domain that initiates nuclear signaling [1,2]. In the case of APP processing, nuclear signaling can be initiated by the production of the intracellular AICD fragment. Once generated by γ-secretase, the AICD fragment can be stabilized and transported to the nucleus by the cytoplasmic adaptor protein Fe65 [14,15]. Upon entering the nucleus the AICD/Fe65 complex can form a tripartite, transcriptionally active complex with the histone acetyltransferase Tip60 [16,17]. Consistent with this model, cells concomitantly over-expressing an APP-Gal4-DNA binding domain fusion protein and Fe65, and carrying a Gal4 UAS-driven reporter construct display a >2000 fold increase in reporter transcription compared to cells over-expressing just the Gal4 DNA binding domain and Fe65 [16]. This increase in transactivation activity is dependent on Tip60 and can be abolished when the interaction between AICD and Fe65 is disrupted by mutagenesis of the AICD NPTY motif, the binding site for Fe65 [16]. However, these data do not rule out a possible effect of full-length APP in inducing nuclear signaling. Indeed, APP nuclear signaling can occur in the absence of γ-secretase activity and therefore does not require the AICD fragment [18]. The relative contribution of AICD-mediated versus holo-APP mediated nuclear signaling is not clear at this time [16-18].

The genomic targets of AICD- or APP-mediated nuclear signaling are not clearly defined. *APP, BACE, Tip60, GSK-3β, Mn-SOD, KAI1*, *NEP *and other genes have all been reported to be targets of APP mediated transcriptional activation [19-22]; however, there is a paucity of confirmatory reports [18]. At this time, the biological role of AICD-mediated transactivation is not clear [20,23,24]. Despite this confusion, evidence suggests that defective APP signaling is involved in AD pathogenesis [25-29].

Given the centrality of APP in AD, it is crucial to identify regulators of APP metabolism, including, but not limited to, APP proteolysis. Regulation of APP metabolism can occur by numerous mechanisms, including regulation of APP transcription, APP translation, APP maturation, intracellular trafficking of full-length APP and APP cleavage products, APP proteolysis, and APP degradation. While Komano and colleagues have used a genetic screen to specifically identify regulators of γ-secretase activity [30], a screen that will identify APP metabolism regulators that act through multiple mechanisms is needed.

Here we describe a novel experimental approach to identify a variety of regulators of APP metabolism. We use an AICD-Gal4 mediated luciferase expression assay as a general reporter of APP metabolism in the human neuroblastoma cell lines, SH-SY5Y. To validate this assay, we utilized pharmacologic agents, as well as forward and reverse genetics, to modulate APP proteolysis, AICD trafficking and AICD transactivation. To determine if regulators of APP maturation and PS1 endoproteolysis also can be detected with this screening approach, we knocked-down Ubiquilin 1 and observed decreased AICD-Gal4 luciferase activity. Using Western blot analysis, we show that Ubiquilin 1 controls APP levels, the ratio of mature to immature APP, as well as presenilin1 endoproteolysis, confirming the previously reported role of Ubiquilin 1 in APP and presenilin1 metabolism in non-neuronal human cell lines [31-34]. Taken together, our results validate the use of the AICD-Gal4 mediated luciferase assay in combination with forward and reverse genetics as a screen to identify APP metabolism regulators.

## Results

### Establishment of a functional assay to identify APP metabolism regulators

**Figure 1:**
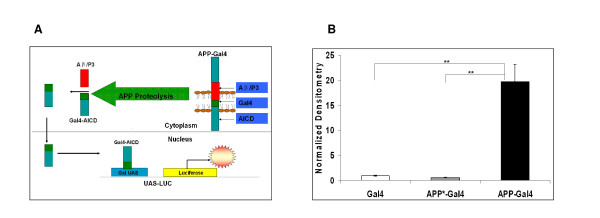
**Functional screen for regulators of APP metabolism**. (A) Model depicting APP-Gal4 reporter system. (B) Firefly luciferase activity is significantly increased in SH-SY5Y cells stably expressing APP-Gal4 and Gal4-UAS Luciferase compared to SY5Y cells stably expressing either Gal4/Gal4-UAS Luciferase or APP*-Gal4/Gal4-UAS Luciferase. Luciferase activity was normalized to total cell number using SYBR Green. Bars represent mean normalized luciferase expression (+/- std. error) of 16 independent trials for each cell line. Student's t-tests with sequential Bonferroni correction for multiple comparisons; ** indicates p < 0.01.

We utilized the APP-Gal4/Gal4-UAS luciferase reporter system (Figure 1A) developed by Cao and Südhof [16]. We established this assay system in our laboratory by creating a SH-SY5Y, human neuroblastoma, cell line that stably expresses the assay components. Three different stable cell lines have been generated; all stably carry a luciferase reporter gene under the control of the Gal4-UAS (Gal4-UAS luciferase). In addition to this reporter gene, one cell line expresses the Gal4 DNA binding domain alone (SY5Y-Gal4), the second cell line expresses APP_695 _fused to the Gal4 DNA binding domain (SY5Y-APP-Gal4), and the third cell line expresses a mutated version of APP_695_-Gal4 (SY5Y-APP*-Gal4). This mutation in APP alters the NPTY motif (P685A; Y687A) and disrupts Fe65 binding to this site [16]. Once these cells were established, luciferase assays were performed to determine the relative luciferase activity of the cell lines (Figure 1B). SY5Y-APP-Gal4 cells have a statistically significant (p < 0.01) ~20 fold increase in luciferase activity compared to SY5Y cells expressing either Gal4 or APP*-Gal4.

### Pharmacologic modulation of α- and γ-secretase activity alters AICD-Gal4 mediated luciferase activity

To determine if monitoring AICD-Gal4 mediated luciferase activity is a valid method to detect alterations in APP metabolism, we used pharmacologic agents known to modulate APP proteolytic processing and compared the effects of these agents on levels of APP proteolytic products and AICD-Gal4 mediated luciferase activity. To accomplish this, we treated our SY5Y-APP-Gal4 cells with pharmacologic modulators of α-, β-, and γ-secretase activity and measured the effects using Western blot analyses for APP cleavage products as well as AICD-Gal4 mediated luciferase activity.

**Figure 2:**
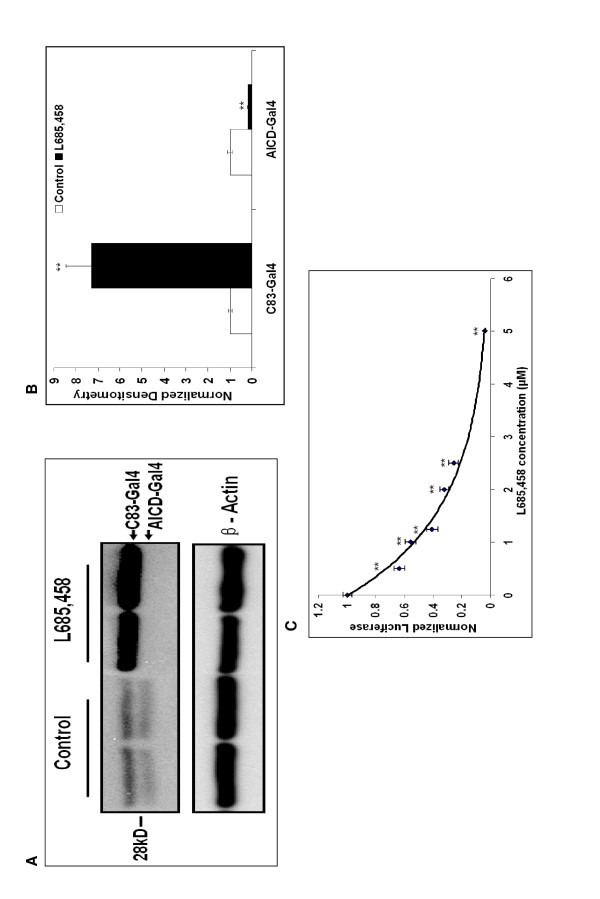
**γ-secretase inhibition decreases AICD-Gal4 levels and AICD-Gal4 mediated luciferase activity in SY5Y-APP-Gal4 cells**. (A) Inhibition of γ-secretase by L-685,458 (5 mM) decreases AICD-Gal4 levels and increases C83-Gal4 levels as detected by Western blot analysis. (B) Quantification of Western blot densitometry in panel A. Normalization for loading differences was achieved by dividing the densitometry values for individual bands by the densitometry values for β-actin in the same lane. (C) Dose-dependent decreases in AICD-Gal4-mediated luciferase activity with increasing concentrations of L-685,458. For the luciferase experiments, points represent mean normalized luciferase activity (+/- standard error) of three independent trials, with luciferase levels normalized to total cell numbers using SYBR Green. Student's t-tests with sequential Bonferroni correction for multiple comparisons was utilized to test for significance. * indicates p < 0.05; ** indicates p < 0.01. "Control" uses the same media as the treatments, and also contains the same amount of DMSO.

L-685,458 is a transition state inhibitor of γ-secretase that prevents Aβ and AICD generation [35]. We treated SY5Y-APP-Gal4 cells with L-685,458 (2.5 μM for 10 hours) or with vehicle (DMSO) and collected cell lysates. We performed Western blot analysis on these cell lysates using an antibody to the C-terminus of human APP. In vehicle treated cells we observe bands migrating at ~28 and ~26 kDa (Figure 2A). Cao and Sudhof observed a similar doublet at approximately the same relative molecular weight. They identified these bands as C83-Gal4 and AICD-Gal4, respectively. In L-685,458 treated cells the intensity of the C83-Gal4 band is significantly increased seven-fold (p < 0.01) and the AICD-Gal4 band is significantly decreased by 80% (p < 0.01; Figure 2B). These results are consistent with the substrate/product relationship between C83-Gal4 and AICD-Gal4. The size difference between C83-Gal4 and AICD-Gal4 is what is expected for γ-secretase cleavage of C83-Gal4. It is also interesting to note that AICD is normally difficult to detect by Western blot analysis, however the AICD-Gal4 fusion levels are quite high. This suggests that AICD-Gal4 catabolism by IDE and/or other proteases is greatly reduced compared to unmodified AICD [36].

L-685,458 treatment of SY5Y-APP-Gal4 cells results in a concentration-dependent decrease in AICD-Gal4 mediated luciferase activity; at a concentration of 2.5 μM there is a ~75% decrease in luciferase activity (Figure 2C). The L-685,458 concentration required for 50% inhibition of AICD-Gal4 mediated luciferase activity is 1.25 μM.

**Figure 3:**
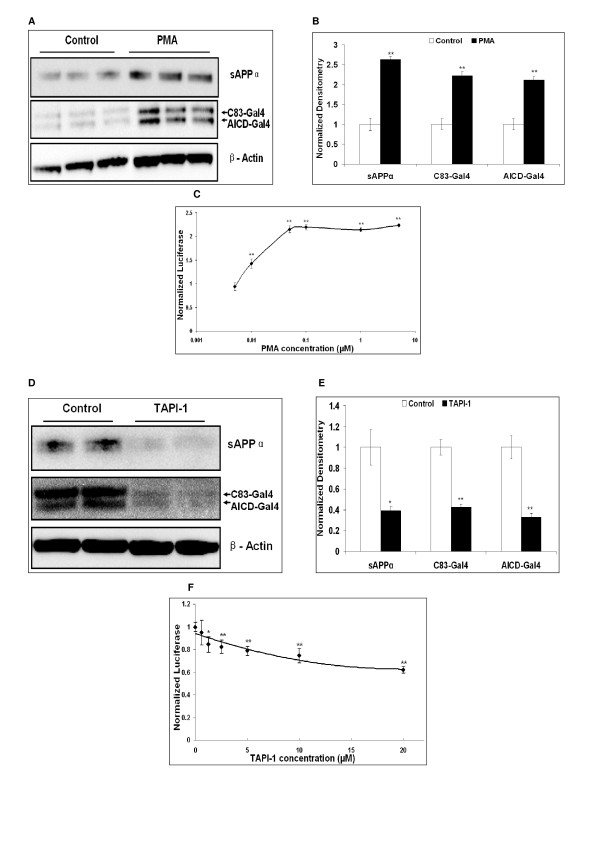
**Pharmacological modulation of α-secretase activity alters AICD-Gal4 levels and AICD-Gal4 mediated luciferase activity in SY5Y-APP-Gal4 cells**. (A) Stimulation of α-secretase by PMA (1 μM PMA for 10 hours) increases sAPPα, C83-Gal4, and AICD-Gal4 levels as detected by Western blot analysis. (B) Quantification of Western blot densitometry in panel A. Normalization for loading differences was achieved by dividing the densitometry values for individual bands by the densitometry values for β-actin in the same lane. (C) Dose-dependent increases of AICD-Gal4-mediated luciferase activity with increasing concentrations of PMA (10 hour incubation). Luciferase levels normalized to total cell number using protein concentration. (D) Inhibition of α-secretases by TAPI-1 (20 μM for two hours) results in decreases in sAPPα, C83-Gal4, and AICD-Gal4 levels as detected by Western blot analysis. (E) Quantification of Western blot densitometry in panel D and normalized β-actin levels in the same lane. (F) Dose-dependent decreases in AICD-Gal4-mediated luciferase activity with increasing TAPI-1concentrations (two hour incubation). For the luciferase experiments, points represent mean normalized luciferase activity (+/- standard error) of three independent trials, with luciferase levels normalized to total cell numbers using SYBR Green. Student's t-tests with sequential Bonferroni correction for multiple comparisons; * indicates p < 0.05; ** indicates p < 0.01. "Control" uses the same media as the treatments and contains the same amount of DMSO as drug treated cells.

The phorbol ester, PMA (phorbol 12-myristate 13-acetate), stimulates α-secretase activity [37]. Treatment of SY5Y-APP-Gal4 cells with PMA (1 μM for 10 hours) resulted in approximately two-fold increase in the levels of the α-secretase cleavage products sAPPα and C83-Gal4 (p < 0.01; Figures 3A &3B). In addition, Western blot analysis revealed a two-fold increase in AICD-Gal4 levels (p < 0.01; Figures 3A &3B). This two-fold increase in AICD-Gal4 levels suggested that a similar PMA-induced increase in AICD-Gal4 mediated luciferase activity should be observed. Indeed, when we measured luciferase activity as a function of increasing PMA concentration (Figure 3C), we observed a dose-dependent increase in luciferase activity. The PMA-induced increases in luciferase activity plateaus at 50 nM PMA. At concentrations of 50 nM and higher, we observed approximately a two-fold increase in AICD-Gal4 mediated luciferase activity in close agreement with the observed two-fold increase in AICD-Gal4 by Western blot analysis.

TAPI-1 (Tumor necrosis factor-α protease inhibitor 1) inhibits α-secretase mediated shedding of the APP ectodomain [38]. Treating the SY5Y-APP-Gal4 cells with TAPI-1 (20 μM for two hours; Figure 3D &3E) resulted in a modest, yet significant decrease of sAPPα (31%, p < 0.05), C83-Gal4 (27%, p < 0.01) and AICD-Gal4 levels (36%; p < 0.01), as well as in AICD-Gal4 mediated luciferase activity (38%; p < 0.01). In addition, TAPI-1 exhibits a dose-dependent effect with 20 μM resulting in a 37% decrease in AICD-Gal4 mediated luciferase activity (Figure 3F). Again, these data show that alterations in AICD-Gal4 levels as detected by Western blot can be accurately detected by the AICD-Gal4 mediated luciferase assay.

Finally, we treated SY5Y-APP-Gal4 cells with a β-secretase inhibitor (β-secretase inhibitor II). This inhibitor prevents BACE-mediated cleavage of APP and generation of Aβ [39]. Treating these cells resulted in no observable change in AICD-mediated luciferase activity. This result is not surprising given the very low levels of β-secretase cleaved APP (C99-Gal4) that we observe in these cells compared to the high levels of α-secretase cleaved APP (C83-Gal4) we observe (Figures 2A, 3A, 3D). We estimate that of all the APP molecules undergoing α- or β-secretase cleavage only about 10% are cleaved by β-secretase, using our Western blot data (data not shown). Therefore, inhibition of BACE, even if effective, may result in an undetectable change in the levels of cleavage products in this experimental scheme.

In summary, pharmacologic modulation of α- and γ-secretase activities alters AICD-Gal4 mediated luciferase activities that accurately correspond to the changes in AICD-Gal4 levels determined by Western blot analysis.

### Genetic manipulation of secretase levels modulates AICD-Gal4 mediated luciferase activity

To further validate the AICD-Gal4 mediated luciferase assay as a reporter of APP metabolism, we over-expressed and knocked-down the expression of genes involved in α-, β- & γ-secretase activities. Again, we compared the effects of over-expression or knock-down on levels of APP proteolytic products quantified by Western blot analysis to AICD-Gal4 mediated luciferase activity in SY5Y-APP-Gal4 cells.

**Figure 4:**
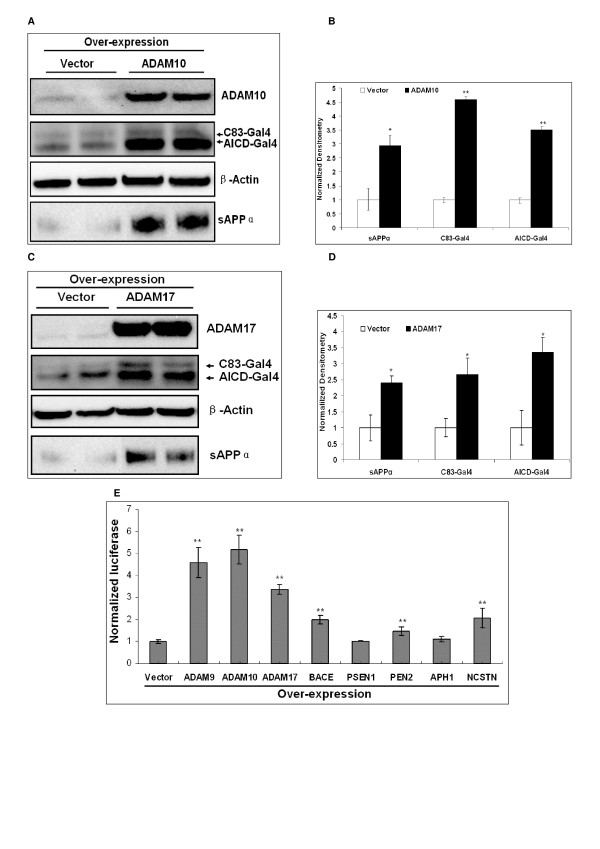
**Over-expression of individual secretase genes in SY5Y-APP-Gal4 cells increases AICD-Gal4 mediated luciferase activity**. (A) Transient over-expression of ADAM10 increases ADAM10, AICD-Gal4, C83-Gal4, and sAPPα levels compared to cells transfected with empty vector. (B) Quantification of Western blot densitometry in panel A. (C) ADAM17 transient over-expression significantly increases ADAM17, AICD-Gal4, C83-Gal4, and sAPPα levels. (D) Quantification of Western blot densitometry in panel C. (E) Transient over-expression of individual secretase genes increases AICD-Gal4 mediated luciferase activity. Luciferase was normalized to transfection efficiency, by dividing by *Renilla *luciferase activity. Individual secretase over-expression plasmids were co-transfected with pRL-SV40 plasmid, expressing *Renilla *luciferase. Bars represent the mean normalized luciferase activity of four independent trials and error bars represent standard errors. Statistical significance was determined using two-sample, one-tail t-tests to compare each secretase gene with the empty vector, followed by sequential Bonferroni procedure to adjust for multiple comparisons. * indicates p < 0.05; ** indicates p < 0.01.

Over-expression experiments were conducted by transiently transfecting individual over-expression plasmids or empty vector controls into the SY5Y-APP-Gal4 cells. Cell lysates and conditioned media were collected 24 – 48 hours post transfection. ADAM10 and ADAM17 over-expression promoted α-secretase cleavage of APP and increased sAPPα secretion as compared to "empty vector" transfected cells (Figure 4A – 4D). ADAM10 and ADAM17 over-expression also significantly increased C83-Gal4 and AICD-Gal4 levels as detected by Western blot (Figure 4A – 4D). Specifically, AICD-Gal4 levels increased approximately three-fold for both (Figure 4B &4D). Measuring AICD-Gal4 mediated luciferase activity, we found that over-expression of ADAM10 and ADAM17 resulted in a statistically significant three to five fold increase in luciferase activity (Figure 4E). Furthermore, over-expression of the β-secretase gene (*BACE*) or individual components of the γ-secretase complex (*PSEN1*, *PEN2*, *APH1*, *and NCSTN*) or another α-secretase member ADAM9 also results in increased luciferase activity (Figure 4E). Specifically, *BACE *over-expression significantly increased luciferase activity approximately two fold (p < 0.01), *PEN2 *and *NCSTN *over-expression increased luciferase activity up to two fold (p < 0.01.) However, over-expression of *PSEN1 *and *APH1 *did not result in any significant change in luciferase activity.

**Figure 5:**
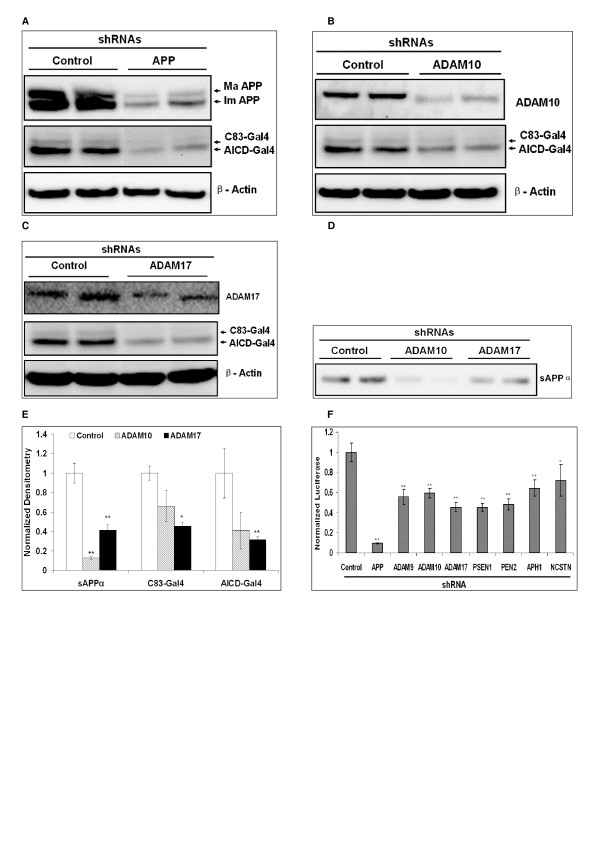
**Knock-down of APP and individual secretase genes in SY5Y-APP-GAL4 cells decreases AICD-Gal4 mediated luciferase activity**. (A) APP-specific shRNA decreases full-length APP, C83-Gal4, and AICD-Gal4 levels compared to the control or "non-silencing" shRNA. Results from duplicate transfections with each shRNA are shown. (B) ADAM10 specific shRNAs decrease endogenous ADAM10, C83-Gal4, and AICD-Gal4 levels compared to the control shRNA. Results from duplicate transfections with each shRNA are shown. (C) ADAM17 specific shRNAs decrease endogenous ADAM17, C83-Gal4, and AICD-Gal4 levels compared to the control shRNA. Results from duplicate transfections with each shRNA are shown. (D) Knock-down of ADAM9, 10, and 17 decrease sAPPα levels compared to control shRNA. (E) Quantification of Western blot densitometries in panels B – D. (F) Transfection with shRNAs specific for APP and individual secretase genes decreases AICD-Gal4-mediated luciferase expression compared to control shRNA. Bars represent the mean normalized luciferase activity of four independent trials and error bars represent standard errors. Statistical significance was determined using two-sample, one tailed t-tests to compare each secretase shRNA with the control shRNA and sequential Bonferroni procedure to adjust for multiple comparisons. * indicates p < 0.05; ** indicates p < 0.01.

We knocked-down the genes responsible for α- and γ-secretase using commercially available shRNAs [40]. A control shRNA, which is not complementary to any known human gene, was used as a negative control. SY5Y-APP-Gal4 cells were transfected with individual shRNAs and selected with 2 μg/ml puromycin for 5 to 7 days. Conditioned media and cell lysates were collected from these cells and utilized for Western blot analyses and luciferase assays. shRNAs specific for APP, ADAM10, and ADAM17 were tested for their ability to knock-down their target genes (Figure 5A, 5B, 5C). Knock-down of these target genes was robust and we have observed significant protein knock-down with at least two different shRNA sequences for each target gene. Consistent with this knock-down of ADAM 10 and ADAM17, sAPPα levels were decreased significantly (Figure 5D &5E). In addition, Western blot analyses showed AICD-Gal4 levels were also decreased when APP, ADAM10 and ADAM17 were knocked-down (Figure 5E). Knock-down of these target genes also decreased AICD-Gal4 mediated luciferase activity (Figure 5F). Specifically, APP knock-down significantly decreased luciferase activity about 80% (p < 0.01); furthermore α-secretase (ADAM10, and ADAM17) knock-down significantly decreased luciferase activity 40–60% (p < 0.01). Individual γ-secretase components, *PSEN1*, *Pen2*, *APH1*, and *NCSTN*, were also knocked-down and this resulted in significant 30–50% decreases in luciferase activity (p < 0.01).

### Genetic manipulation of Fe65 and Tip60 levels modulates AICD-Gal4 mediated luciferase activity

**Figure 6:**
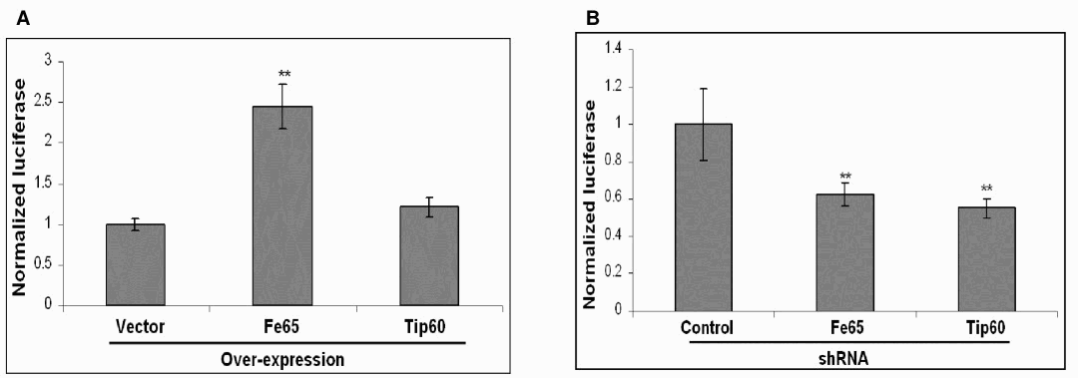
**Genetic alteration of Fe65 or Tip60 levels modulates AICD-Gal4 mediated luciferase activity**. (A) Transient over-expression of Tip60 or Fe65 in SY5Y-APP-Gal4 cells increases AICD-Gal4 production compared to empty vector controls. (B) Knock-down of Fe65 or Tip60 in SY5Y-APP-Gal4 cells decreases AICD-Gal4 mediated luciferase activity Bars represent the mean normalized luciferase activity of four independent trials and error bars represent standard errors. Statistical significance was determined using two-sample, one-tailed t-tests to compare each secretase gene and "vector" or "control" and sequential Bonferroni procedure to adjust for multiple comparisons. * indicates p < 0.05.

To determine if changes in AICD metabolism modulated AICD-Gal4 mediated luciferase activity, we over-expressed and knocked-down Fe65 and Tip60. Transient over-expression of Fe65 significantly increased luciferase activity more than two-fold (p < 0.01), while transient over-expression of Tip60 resulted in a 30% increase in luciferase activity that was not significant (Figure 6A). Knock-down of Fe65 and Tip60 resulted in a significant 40–50% decrease in luciferase activity (Figure 6B; p < 0.01).

### Ubiquilin 1 regulates AICD-Gal4 levels

Having shown that monitoring AICD-Gal4 mediated luciferase activity accurately measures changes in AICD-Gal4 levels induced by changes in secretase activity/levels, we wanted to determine if this approach could detect regulators with a less direct role in APP proteolysis and AICD signaling. We decided to test Ubiquilin 1 because (i) the gene encoding Ubiquilin 1 (*UBQLN1*) is located in a region of chromosome 9 that displays linkage to AD in several independent samples [41-46], (ii) a polymorphism in *UBQLN1 *modulates AD risk in several independent samples [47-49], (iii) Ubiquilin 1 can modulate APP trafficking to the cell surface in HEK-293 and H4 cell lines [31], and (iv) Ubiquilin 1 can modulate γ-secretase activity, though the consequences of this modulation on γ-secretase substrates were not determined [32-34]. Given this, testing Ubiquilin 1 would determine if our genetic screen can detect regulators of APP trafficking and presenilin endoproteolysis. Furthermore, the role of Ubiquilin 1 in APP metabolism regulation has not been previously investigated in SH-SY5Y cells.

**Figure 7:**
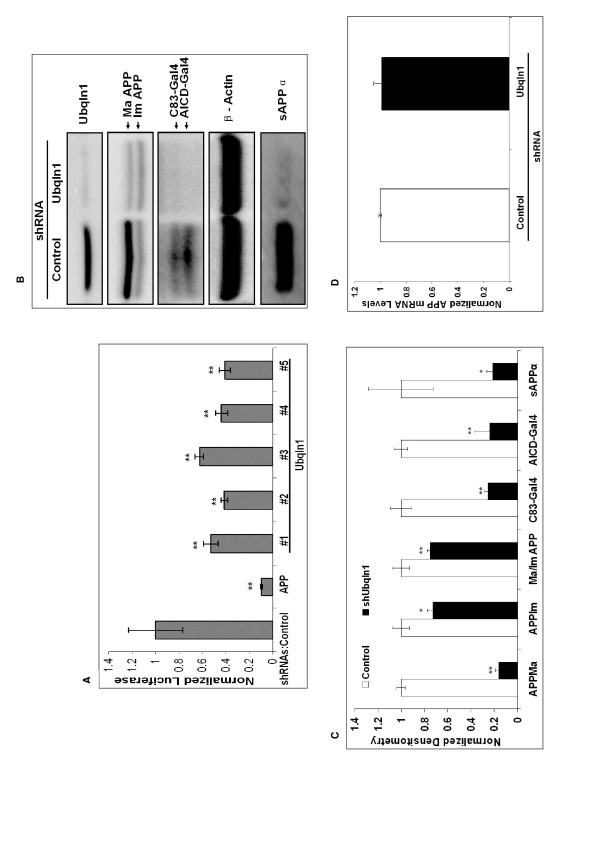
**Ubiquilin 1 knock-down regulates APP-Gal4 metabolism in SY5Y-APP-Gal4 cells**. (A) Ubiquilin 1 knock-down decreases AICD-Gal4-mediated luciferase activity. SY5Y-APP-Gal4 cells stably expressing the control shRNA, an APP specific shRNA, or five different shRNA targeting Ubiquilin 1 were generated. Cell lysates were utilized to measure AICD-Gal4 mediated luciferase activity. Bars represent the mean normalized luciferase activity (+/- standard error) of six independent trials. (B) SY5Y-APP-Gal4 cells stably expressing Ubiquilin 1 specific shRNA (#2) have decreased Ubiquilin 1, mature and immature APP-Gal4, C83-Gal4, AICD-Gal4, and sAPPα levels compared to cells expressing control shRNA. (C) Quantification of Western blot results. Bars represent mean densitometry (+/- standard error) of three independent trials. Black bars represent the densitometry from Ubiquilin 1 knock-down cells; white bars represent the densitometry from cells expressing control shRNA. Abbreviations: Ma APP denotes mature APP, Im APP denotes immature APP; Ma/Im APP denotes the mature APP/immature APP ratio. Statistical significance between mock and over-expression for each measure was determined using a two-sample, one tailed t-test and sequential Bonferroni procedure to adjust for multiple comparisons. * indicates p < 0.05; ** indicates p < 0.01. (D) Ubiquilin 1 knock-down does not alter APP mRNA levels compared to control shRNA as measured by quantitative PCR.

**Figure 8:**
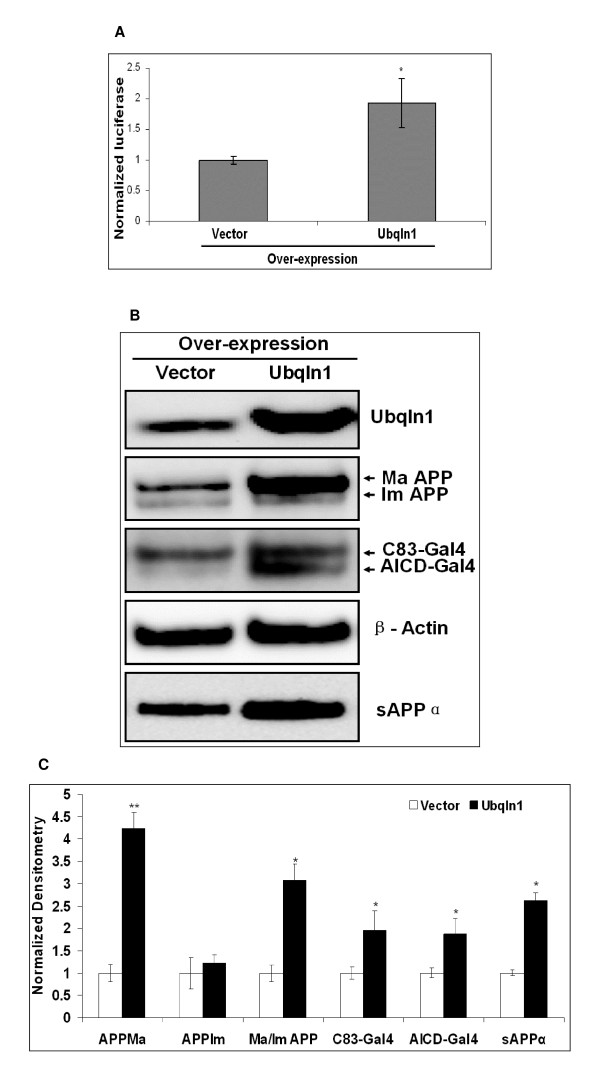
**Ubiquilin 1 over-expression regulates APP-Gal4 metabolism in SY5Y-APP-Gal4 cells**. (A) Transient Ubiquilin 1 over-expression increases AICD-Gal4 mediated luciferase activity. SY5Y-APP-GAL4 cells were transiently co-transfected with *UBQLN1 *over-expression plasmid and a *Renilla *luciferase over-expression plasmid (pRL-SV40). The latter was used as a transfection efficiency control to normalize AICD-Gal4 mediated luciferase activity. Bars represent the mean normalized luciferase activity (+/- standard error) of six independent trials. Statistical significance was determined using two-sample, one-tailed t-tests to compare each experimental shRNA to the control shRNA and sequential Bonferroni procedure to adjust for multiple comparisons. (B) SY5Y-APP-Gal4 cells transiently over-expressing Ubiquilin 1 have increased Ubiquilin 1, mature and immature APP-Gal4, C83-Gal4, AICD-Gal4, and sAPPα levels compared to vector only cells. (C) Quantification of Western blot results. Bars represent mean densitometry (+/- standard error) of three independent trials. Black bars represent the densitometry from Ubiquilin 1 over-expressing cells; white bars represent the densitometry from cells expressing empty vector control. Abbreviations: Ma APP denotes mature APP, Im APP denotes immature APP; Ma/Im APP denotes the mature APP/immature APP ratio. Statistical significance between mock and over-expression for each measure was determined using a two-sample, one tailed t-test and sequential Bonferroni procedure to adjust for multiple comparisons. * indicates p < 0.05; ** indicates p < 0.01.

SY5Y-APP-Gal4 cells were transfected separately with five different Ubiquilin 1 shRNAs, APP shRNA and the control shRNA. Cell lysates were collected and utilized for luciferase assays. Individually, all five Ubiquilin 1 shRNA constructs significantly decreased luciferase activity. They resulted in 50% (p < 0.01), 60% (p < 0.01), 40% (p < 0.01), 60% (p < 0.01), and 60% (p < 0.01) decreases in luciferase activity, respectively (Figure 7A) as compared to cells expressing the control shRNA. To confirm the role of Ubiquilin 1 in AICD-mediated transcriptional activity suggested by these results, we transiently over-expressed Ubiquilin 1 in SY5Y-APP-Gal4 cells and measured luciferase activity. We observed that Ubiquilin 1 over-expression resulted in an approximately 90% (p < 0.05) increase in luciferase activity compared to the empty vector control (Figure 8A).

### Ubiquilin 1 regulates APP and PS1

To begin to gain insight into the mechanism(s) by which Ubiquilin 1 modulates AICD-Gal4 mediated luciferase expression, we utilized Western blot analysis of cell lysates and conditioned media from SY5Y-APP-Gal4 cells in which Ubiquilin 1 was knocked-down or over-expressed to monitor APP and Ubiquilin 1 metabolism. Specifically, we analyzed cell lysates and conditioned media of cells expressing Ubiquilin 1 shRNA number 2, since transfection with this shRNA led to the largest decrease in luciferase activity. Expression of this shRNA resulted in a robust Ubiquilin 1 knock-down and led to significantly decreased levels of mature full-length APP, immature full-length APP, AICD-Gal4, C83-Gal4, and sAPPα (Figures 7B &7C). To determine if Ubiquilin 1-induced changes in APP mRNA levels underlie the observed changes in full length APP levels, we performed real-time, quantitative PCR on SY5Y-APP-Gal4 cells stably expressing either control or Ubiquilin 1 shRNAs. No Ubiquilin 1-induced changes in APP mRNA levels were observed (Figures 7D). This suggests that Ubiquilin 1 regulation of full-length APP levels occurs post-transcriptionally.

We studied Ubiquilin 1 over-expression to determine if we observed the converse effects on APP proteolytic products and full-length APP. Indeed, we observed that Ubiquilin 1 over-expression resulted in increased levels of mature and immature full-length APP, AICD-Gal4, C83-Gal4, and sAPPα (Figures 8B &8C).

The Ubiquilin 1-induced effects on full-length APP are greater on mature APP levels than on immature APP levels (Figure 7C &8C). This results in a decrease in the ratio of mature to immature full-length APP-Gal4 when Ubiquilin 1 is knocked-down (p < 0.01; Figure 7C) and an increase in this ratio when Ubiquilin 1 is over-expressed (p < 0.05; Figure 8C).

**Figure 9:**
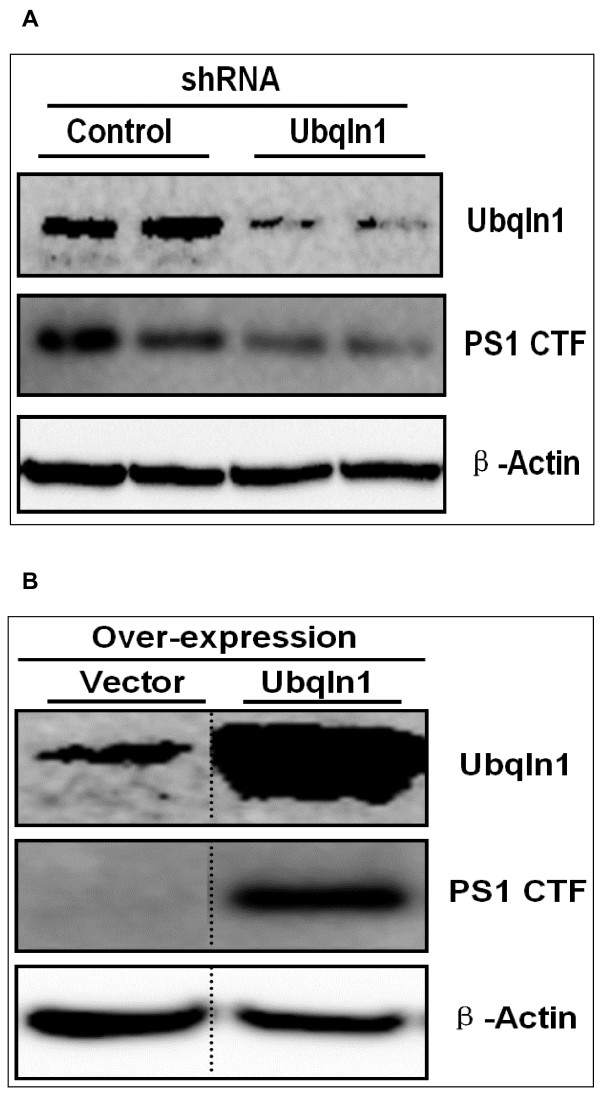
**Ubiquilin 1 regulates Presenilin 1 endoproteolysis in SY5Y-APP-Gal4 cells**.(A) Ubiquilin 1 knock-down decreases PS1 CTF levels in SY5Y-APP-Gal4 cells. (B) Ubiquilin 1 over-expression increases PS1 CTF levels in SY5Y-APP-Gal4 cells.

Since Ubiquilin 1 has been reported to regulate PS1 endoproteolysis in HEK-293 cell lines, we sought to determine if the Ubiquilin 1-induced changes that we observed in AICD-Gal4 and C83-Gal4 levels may be due in part to changes in PS1 endoproteolysis [34]. Ubiquilin 1 knock-down in SY5Y-APP-Gal4 cells decreased PS1 carboxy-terminal fragment levels (PS1-CTF; Figure 9A) and Ubiquilin 1 over-expression increased PS1-CTF levels (Figure 9B). We did not observe any changes in the levels of ADAM 10, ADAM 17 or BACE when Ubiquilin 1 was over-expressed or knocked-down (data not shown).

Finally, we over-expressed Ubiquilin 1 in naïve SH-SY5Y to ensure that the results we observed are not limited to the SY5Y-APP-Gal4 cell line. We found that in these naïve cells Ubiquilin 1 over-expression resulted in increased total, mature and immature APP, sAPP as well as PS1 CTF, consistent with our findings in SY5Y-APP-Gal4 cells (data not shown).

## Discussion

Taking advantage of the APP intracellular domain's (AICD) ability to activate transcription, we established an assay to monitor APP metabolism in the human neuroblastoma cell line, SH-SY5Y. We are using this assay in combination with RNAi-mediated knock-down of positional candidate genes as a genetic screen to identify regulators of APP metabolism. Here we describe validation of this experimental approach using pharmacologic and genetic modulation of known APP metabolism regulators. We find that AICD-Gal4 mediated luciferase activity is significantly and accurately changed when secretases, Fe65, Tip60, or Ubiquilin 1 levels/activities are modulated pharmacologically or genetically. The ability of Ubiquilin 1 to regulate APP metabolism in SH-SY5Y cells had not been investigated previously. Our initial findings show that in these cells Ubiquilin 1 regulates total APP levels, APP maturation and PS1 endoproteolysis. Our results lead us to conclude that the genetic screen we describe is capable of identifying genes that encode regulators of APP proteolysis, APP maturation, APP levels, and AICD activity.

### Validation of AICD-Gal4 luciferase assay

The functional assay for identifying APP metabolism regulators relies on the ability of an AICD-Gal4 fusion to transactivate a firefly luciferase reporter gene [16]. While the biological role of AICD-mediated transactivation is unclear [20,23,24,50], we utilized this transactivation function purely as a reporter of APP processing and therefore APP metabolism. We determined that monitoring AICD-Gal4 mediated luciferase activity is correlated to AICD-Gal4 levels by utilizing pharmacologic and genetic agents to regulate secretase activities and thereby modulate AICD-Gal4 levels. In SH-SY5Y cells stably expressing an APP-Gal4 fusion protein and a Gal4-UAS driven luciferase reporter construct (SY5Y-APP-Gal4 cells), we utilized TAPI-1 and L-685,458 to inhibit α- and γ-secretases respectively. TAPI-1 inhibits α-secretase cleavage of APP as well as several other cell surface proteins including TNFα [38]. L-685,458 is a potent and selective cell-permeable γ-secretase inhibitor [51]. Both of these inhibitors decreased AICD-Gal4 levels and decreased AICD-Gal4 mediated luciferase activity to similar levels. Inhibiting BACE activity did not have an appreciable effect on AICD-Gal4 levels or AICD-Gal4 mediated luciferase activity, which is not surprising since the majority of APP processing occurs via the α-secretase pathway in SH-SY5Y cells. Stimulation of α-secretase using PMA [52] increased AICD-Gal4 levels and increased AICD-Gal4 mediated luciferase activity to similar levels.

To further validate our functional assay, we over-expressed and knocked-down genes that encode the α-, β-, and γ-secretases. Similar to the effects of pharmacologic modulators of secretases, over-expressing or knocking-down secretase genes resulted in predictable alterations in AICD-Gal4 levels as measured by Western blot analysis. The changes in luciferase activity induced by secretase over-expression or knock-down mirrored the trends observed in the Western blot analysis. Knock-down of APP had the most dramatic effect on AICD-Gal4 mediated luciferase activity while knock-down of genes encoding α- and γ-secretases resulted in significant decreases in AICD-Gal4 mediated luciferase.

To assess the quality of the AICD-Gal4 mediated luciferase assay we calculated the "Z-factor" for the assay in response to the known APP metabolism modulators[53]. The Z-factor is a dimensionless metric that takes assay dynamic range and data variation into consideration to assess the utility and reliability of the assay. Scores between 0.5 and 1.0 indicate an excellent assay [53]. Using the data we collected, we calculated Z-factors for pharmacologic and genetic modulation of the secretases (Table 1). For all of these conditions we obtain Z values between 0.5 and 1.0, indicating that our experimental approach is robust and has the capability of identifying APP metabolism regulators that increase or decrease AICD generation.

**Table 1:** Z-factor values

**APP Metabolism Modulators**		**Z factor**
Pharmacological	PMA	0.77
	TAPI-1	0.63
	L685,458	0.74
shRNA	APP	0.60
	ADAM10	0.71
	ADAM17	0.70
Over-expression	ADAM10	0.72
	ADAM17	0.60

Z-factor values for AICD-Gal4 Luciferase assay calculated when APP metabolism is modulated by pharmacologic or genetic approaches.

### AICD metabolism regulators modulate AICD-Gal4 luciferase activity

AICD-Gal4 mediated transactivation has been shown to require Fe65 and Tip60. Fe65 is an adaptor protein that binds to the NPTY sequence in AICD and mediates intracellular trafficking of AICD-Gal4 from the cytoplasm into the nucleus [16]. Once inside the nucleus, the AICD-Gal4/Fe65 complex recruits the histone acetyltransferase, Tip60. Fe65 and Tip60 are both required for AICD-Gal4 transactivation activity. We observed increased AICD-Gal4 mediated luciferase activity when we over-expressed Fe65 or Tip60 and decreased luciferase activity when either of these genes was knocked-down.

### Ubiquilin 1 modulates APP metabolism in SH-SY5Y cells

Having validated our experimental approach using direct regulators of APP proteolysis and AICD-metabolism, we then sought to determine if Ubiquilin 1 could modulate AICD-Gal4 mediated luciferase activity. Ubiquilin 1 has been shown to regulate presenilin1 endoproteolysis and APP trafficking in HEK-293 cells [31-34] and therefore testing Ubiquilin 1 would help to determine whether our experimental approach could detect APP metabolism regulators that are not directly involved in APP proteolysis nor in AICD signaling. When Ubiquilin 1 was knocked-down, AICD-Gal4 luciferase activity was significantly decreased.

Ubiquilin 1 is a conserved protein that contains an NH_2_-terminal ubiquitin-like domain (UBL) and a COOH-terminal ubiquitin-associated (UBA) domain [32]. Through these domains, Ubiquilin 1 associates with ubiquitin ligases and the proteosome and is proposed to link ubiquitination with proteosome-mediated protein degradation. This suggests that Ubiquilin 1 plays a role in responding to protein misfolding, aggregation, and/or stress [32,54]. In the brains of AD patients, there is increased Ubiquilin 1 in neurons containing neurofibrillary tangles (NFTs), as compared to control brains [32]. In the brains of Parkinson's disease patients, as well as patients with diffuse Lewy body disease (DLBD), there is strong Ubiquilin 1 staining of Lewy bodies [32]. Finally, a polymorphism in the *UBQLN1 *gene has been shown to increase AD risk in family-based and large case-control samples [47-49].

The role of Ubiquilin 1 in AD pathogenesis may be due to its ability to regulate formation of active γ-secretase complexes and/or regulate APP trafficking [31-34]. Monteiro and colleagues have found that Ubiquilin 1 can regulate full-length Presenilin1 (PS1), Presenilin2 (PS2), Nicastrin, and PEN-2 levels as well as PS1 and PS2 endoproteolysis [32-34]. Specifically, Ubiquilin 1 over-expression increased full-length presenilin (PS1 and PS2) levels in HeLa cells. In HEK-293 cells, Ubiquilin 1 over-expression decreased presenilin endoproteolysis while Ubiquilin 1 knock-down increased presenilin endoproteolysis [34]. In addition, Monteiro and colleagues show that nicastrin and Pen-2 levels are decreased by Ubiquilin 1 over-expression and increased by Ubiquilin 1 knock-down in HEK-293. In addition to these effects on γ-secretase components, Hiltunen and colleagues reported that Ubiquilin 1 knock-down decreased steady-state full-length immature APP levels, increased trafficking of APP from intracellular compartments to the cell surface, and increased steady-state sAPPα levels in HEK-293 and H4 cell lines [31]. These effects on APP levels and secretion altered Aβ40 and Aβ42 levels. However, Ubiquilin 1 knock-down did not alter α-, β-, or γ-secretase levels or C83 and C99 levels in these cell lines.

Here we found that in the human neuroblastoma cell line, SH-SY5Y, Ubiquilin 1 regulates total full-length APP, the ratio of mature to immature APP, as well as PS1 endoproteolysis. To arrive at these conclusions, we over-expressed and knocked-down Ubiquilin 1 in SY5Y-APP-Gal4 cells and monitored APP metabolism using Western blot analysis. We found that Ubiquilin 1 knock-down decreased levels of AICD-Gal4, C83-Gal4, sAPPα, full-length mature and immature APP, and the ratio of mature to immature APP. Ubiquilin 1 over-expression elicited the opposite effect on the levels of these molecules.

The fact the ratio of mature to immature APP is altered by Ubiquilin 1 in the absence of APP mRNA level changes suggests that Ubiquilin 1 modulates trafficking through the secretory pathway in SH-SY5Y cells. This conclusion was reach by Hiltunen and colleagues when investigating the role of Ubiquilin 1 on APP metabolism in H4 and HEK-293 cell lines [31].

Given the existing reports that Ubiquilin 1 regulates PS1 levels and endoproteolysis in HeLa and HEK-293 cells, respectively, we sought to determine if the observed changes in APP processing may be due, in part, to Ubiquilin 1 mediated changes in PS1 metabolism [32-34]. Interestingly, Hiltunen and colleagues did not observe any changes in PS1 levels or endoproteolysis upon transient Ubiquilin 1 knock-down in HEK-293 [31]. In SY5Y-APP-Gal4 cells, we observed that Ubiquilin 1 knock-down decreases PS1 endoproteolysis and Ubiquilin 1 over-expression promotes PS1 endoproteolysis. Presumably these changes in PS1-CTF levels alter γ-secretase activity and cleavage of other γ-secretase substrates. At this time, it is not clear how Ubiquilin 1 regulates PS1 endoproteolysis. No alterations in ADAM10, ADAM17, or BACE levels were observed when Ubiquilin 1 was knocked-down or over-expressed. These results suggest that Ubiquilin 1 regulates APP metabolism not only by controlling the ratio of mature to immature APP but also by post-transcriptionally controlling total APP (mature and immature) levels and PS1 endoproteolysis.

It is interesting to note that the effects of Ubiquilin 1 over-expression/knock-down on APP and presenilin metabolism that we observe in SH-SY5Y cells are different than those observed in HEK-293 and HeLa cells [31,34]. In SH-SY5Y cells we find Ubiquilin 1 knock-down decreased total, mature, and immature full-length APP, sAPPα, C83 and AICD steady-state levels and the ratio of mature to immature APP, while over-expression increased these same steady-state levels. In addition, Ubiquilin 1 over-expression increased PS1 endoproteolysis. In HEK-293 cells, Hiltunen et al. found that Ubiquilin 1 knock-down decreased steady-state immature full-length APP levels, increased sAPPα levels, and no effects were observed in C83, C99, AICD, and PS1 CTF levels [31]. However, Massey et al. observed an increase in PS1 endoproteolysis in HEK-293 cells [34]. In SH-SY5Y cells however, Ubiquilin 1 seemingly has opposite effects on APP and presenilin metabolism than observed in HEK-293. At this time the reasons for these differences are not clear; they could be due to differences in experimental procedure (e.g. differences in cell confluency, and/or RNAi techniques [transient siRNA versus stable shRNA]) and/or inherent differences in these two cell types. One of the noticeable differences between these cells is that in SH-SY5Y cells, the majority of full-length APP is mature, whereas in HEK-293 the majority of full-length APP is immature (data not shown). Cell type dependent effects of Ubiquilin 1 have been observed previously. In COS7 cells, Ubiquilin 1 over-expression reduced cell surface expression of nicotinic acetylcholine receptors (nAChRs), while in superior cervical ganglion neurons Ubiquilin 1 over-expression had no effect on nAChR levels [55]. These cell type-dependent effects are interesting given the differential vulnerability observed in AD brains, where subsets of neocortical and hippocampal neurons preferentially degenerate [56]. In addition to these cell type-dependent effects, Ubiquilin 1 has been shown to function in seemingly opposite ways. Ubiquilin 1 over-expression has been shown to promote accumulation of some proteins [HASH-1[57], HES-1[57], and GABA_A _receptor[58]] as well as to promote degradation of other proteins [nAChRs[55] and Hepatitis C virus RNA-dependent RNA polymerase [NS5B][59]]. It will be important to study the role of Ubiquilin 1 on APP metabolism in primary neurons and *in vivo *to determine its true role in regulating APP metabolism and in AD pathogenesis.

Our Ubiquilin 1 results suggest that in SH-SY5Y cells, Ubiquilin 1 regulates APP metabolism not only by controlling the ratio of mature to immature APP but also by post-transcriptionally controlling total APP (mature and immature) levels and PS1-CTF levels.

### AICD-Gal4 luciferase assay accurately reports AICD-Gal4 levels

**Figure 10:**
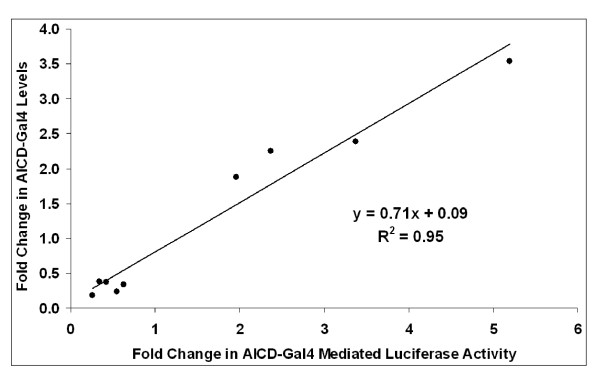
**Correlation between AICD-Gal4 mediated luciferase levels and AICD-Gal4 levels determined by Western blot analysis**. Using data from pharmacologic (PMA, TAPI-1, L-685,458), over-expression (ADAM 10, ADAM17, Ubiquilin 1) or knock-down (ADAM 10, ADAM17, Ubiquilin 1) mediated modulation of AICD-Gal4 levels we plotted the average fold change in AICD-Gal4 levels versus the average fold change in AICD-Gal4 mediated luciferase activity. For transient over-expression, luciferase activity and AICD-Gal4 levels were normalized to transfection efficiency by Renilla luciferase activity assays. The line is represents the least squares linear regression to this data.

Finally, we were struck by the ability of the AICD-Gal4 mediated luciferase assay to accurately report AICD-Gal4 levels. To determine if these measures of AICD-Gal4 were correlation, we plotted the change in luciferase activity versus the change in AICD-Gal4 levels as measured by Western blot analysis. AICD-Gal4 levels were modulated by pharmacologic or genetic modulation of secretases and Ubiquilin 1. This analysis revealed a strong and significant correlation between AICD-Gal4 levels and AICD-Gal4-mediated luciferase expression (Figure 10; R^2 ^= 0.95; p = 6 *10^-6^). The best fit line of this relationship has a slope close of 0.71 demonstrating that this luciferase assay provides an accurate reporter of changes in AICD-Gal4 levels. In addition, measuring AICD-Gal4 mediated luciferase activity provides a simple, quick and inexpensive means for monitoring changes in APP metabolism. The genetic screen we describe can be successfully utilized to identify genes that putatively modulate AICD-Gal4 levels. Additional assays, including as Western blot and ELISA, will be necessary to confirm their role in APP metabolism regulation and gain insight into the mechanism of regulation.

## Conclusion

We have established and validated an AICD-Gal4 based functional assay in SH-SY5Y cells. Using this assay in combination with RNAi, we have developed a genetic screen to identify regulators of APP metabolism. This screen accurately, robustly, and easily measures changes in AICD-Gal4 levels. We demonstrate that these AICD-Gal4 levels can be altered by pharmacologic or genetic modulation of genes that directly regulate APP levels, AICD trafficking/signaling, APP maturation, and APP proteolysis. Using this approach, we show that Ubiquilin 1 can regulate AICD-Gal4 levels in SH-SY5Y cells. Ubiquilin 1 regulates AICD-Gal4 levels by modulating APP levels, the ratio of mature to immature APP, and PS1 endoproteolysis. Taken together, our results demonstrate that this genetic screen is capable of identifying APP metabolism regulators that can modulate the APP proteolytic processing, APP maturation, APP levels, and AICD trafficking/signaling.

## Methods

### Chemicals and antibodies

Phorbol 12-myristate 13-acetate (PMA), L-685,458, and puromycin were purchased from Sigma. TAPI-1 was purchased from Peptides International. β-secretase inhibitor II, N-Benzyloxycarbonyl-Val-Leu-leucinal Z-VLL-CHO, was purchased from Calbiochem. The APP C-terminal antibody (A8717; 1:1000) and β-actin antibody (1:10,000) were purchased from Sigma. The 6E10, anti-APP antibody was purchased from Covance and utilized for detection of sAPPα (1:1000). The BACE1 antibody (1:1000) was purchased from Bioscience. The ADAM10 (C-terminal) antibody (1:1000) was purchased from ProSci. The Ubiquilin 1 antibody (1:160) was purchased from Zymed. The ADAM17 antibody (1:1000) was purchased from Chemicon. The HRP-conjugated secondary antibodies (anti-mouse and anti-rabbit) (1:10,000) were purchased from GE.

### Plasmids

The plasmids APP-Gal4, APP*-Gal4 and Gal4, Gal4-UAS-luciferase (encoding firefly luciferase) were kindly provided from Dr. Thomas Südof, and are described elsewhere [16]. Briefly, each of these plasmids encodes only the DNA binding domain of Gal4. The ADAM10 over-expression plasmid was kindly provided by Dr. Paul Saftig. The ADAM9 and ADAM17 were provided by Dr. Carl Blobel. The empty vector of ADAM9, ADAM10 and ADAM17 is pcDNA3.1. Ubiquilin 1 over-expression plasmid, which was constructed from pCMV vector, was kindly provided by Dr. Mervyn J. Monteiro.

### Cells and cell culture

SH-SY5Y and naïve human embryonic kidney (HEK)-293 cells were purchased from ATCC. These cell lines were cultured in Dulbecco's modified Eagle's medium (DMEM) supplemented with 10% fetal bovine serum, 2 mM L-glutamine, 100 units/ml penicillin, and 100 μg/ml streptomycin. SH-SY5Y cells that stably express APP-Gal4, APP*-Gal4, or Gal-4, and carrying the Gal4-UAS-luciferase reporter construct were constructed by co-transfecting one of the Gal4 constructs, the pCDNA3.1 plasmids, along with the Gal4-UAS plasmid, and selecting resistant clones with 400 μg/ml G418. These cells were then tested for γ-secretase dependent luciferase activity. Clonal lines that stably express luciferase were obtained and were maintained with media containing 200 μl/ml G418.

### RNAi

Plasmid-based shRNA constructs were purchased from Open-Biosystems (Birmingham, AL). These constructs are part of the human retroviral shRNA library housed at the Drexel University RNAi Resource Center. We utilized the following target specific shRNAs: for UBQLN1 shRNAs (Open Biosystems catalog #s: v2HS_58534, v2HS_254856, v2HS_254715, v2HS_255129 and v2HS_58531); for ADAM9 shRNAs (V2HS_17130, V2HS_17127, V2HS_17126, and V2HS_17129); for ADAM10 shRNAs (v2HS_94294, v2HS_94297, and v2HS_94295); for ADAM17 shRNAs (RHS3979-9619367, RHS3979-9619368, RHS3979-9619369, and RHS3979-9619370); for BACE1 shRNAs (V2HS_25207, V2HS_25209, V2HS_25206, V2HS_25205, V2HS_25210); for PSEN1 shRNAs (v2HS_89932, v2HS_89931); for PSEN2 shRNAs (v2HS_93093); for APH1 shRNAs(v2HS_117094, v2HS_117096); for NCSTN shRNAs(v2HS_255892). As a negative control shRNA, we utilized the non-silencing shRNA from Open Biosystems, Inc. (RHS 1707). shRNA constructs were transfected using Arrest-In transfection reagent (Open Biosystems, Inc.) using the conditions suggested by the manufacturer. Stably expressing shRNA clones were generated by adding 2 μg/ml puromycin 24 hours post-transfection. Populations of resistant clones were detected five to seven days post-transfection.

### Western Blot Analysis

Cells were lysed in RIPA cell lysis buffer (50 mM Tris-HCL pH 7.4, 150 mM NaCl, 1 mM EDTA, 1% NP-40, 1 mM PMSF and 1 μg/ml aprotinin, 1 μg/ml leupeptin, and 1 μg/ml pepstatin), and centrifuged at 14,000 rpm for 15 minutes at 4°C. The resulting supernatant was transferred to a new micro-centrifuge tube. The protein concentration of the cell lysates was determined using the BCA protein assay kit (Pierce, Rockford, IL) according to the manufacturer's instructions. Equal quantities of protein were loaded into the wells of 4–12% Bis-Tris polyacrylamide gels (Invitrogen) along with See Blue plus 2 protein marker (Invitrogen). Gels were run using MES running buffer and transferred to PVDF membrane (Immobilon P^SQ^, Millipore) using a semi-dry transfer apparatus (Owl Scientific) and NuPage transfer buffer (Invitrogen). PVDF membranes were blocked in TBST with 5% dry milk for at least two hours, washed extensively, then incubated with primary antibody for either one hour at room temperature or overnight at 4°C. After removing the primary antibody, membranes were extensively washed and incubated with either goat-anti-rabbit-HRP or goat-anti-mouse-HRP secondary antibodies (1:10,000; GE) for one hour at room temperature. Membranes were washed and developed using West Dura Extended Duration Substrate (Pierce). The blot was visualized using a FluoroChem 8900 imaging system (Alpha Innotech), and signals were quantified using AlphaEase Fc software. To account for any differences in loading, target band densitometries were divided by actin densitometries obtained from the same lane. These corrected densitometries were normalized to controls in each experiment.

Detection of sAPPα followed the protocol detailed in Lanni et al. and Bergamaschi et al. [52,60]. Briefly, conditioned media was collected and 48% trichloroacetic acid (TCA) was added so that the TCA final concentration was 15%. This mixture was incubated on ice for 30 minutes, and centrifuged at 14,000 rpm for 20 minutes. Following this spin, the supernatant was aspirated and discarded. 500 μl of ice cold acetone was used to resuspend the pellet. This mixture was placed at -20°C for at least 30 minutes, followed by centrifugation at 14,000 rpm for 20 minutes. The supernatant was carefully aspirated and discarded. The remaining pellet was air dried for 10 minutes and then 20 μl of RIPA was added and this sample was utilized for Western blot analysis. The sAPPα bands were detected using the 6E10 (1:1000) as the primary antibody.

### Luciferase assays

For firefly luciferase assays, 7,500 cells were plated into the 96 well plates. In an experiment, each treatment was applied to a total of six wells. After treatments, conditioned media was aspirated and discarded. 100 μl GLB (Glo Lysis Buffer, Promega) was added to lyse the cells. 30 μl of each cell lysates was transferred to a white plate (Greiner Bio-one), and 30 μl Steady-Glo (Promega) was added. Luciferase was measured using a Top-Count Scintillation Counter/Luminescence Reader (Packard, Inc.) Another 30 ul of each cell lysates was transferred to the other white color plate, and 30 μl 20× SYBR Green (diluted in PBS from Invitrogen 10,000× SYBR Green) was added. SYBR Green fluorescence was measured after 5 minutes incubation in dark using an excitation wavelength of 485 nm, and emission wavelength of 527 nm, and an integration time of 0.1 seconds on a Fluoroscan Ascent FL fluorescence plate reader (Thermo Labsystems, Inc.). The luciferase signal was normalized to cell number by dividing the luciferase signal by the SYBR Green reading for the same well. For dual luciferase assays, SH-SY5Y-APP-Gal4 cells that stably express firefly luciferase were co-transfected with pRL-SV40, which constitutively over-expresses *Renilla *luciferase, along with other plasmids. The dual luciferase assay was performed 24 – 48 hours post-transfection. The media was aspirated and discarded. 30 μl Dual-Glo luciferase substrate (Promega) was added to lyse the cells. All cell lysates were resuspended and transferred to a white 96 well plate (Greiner Bio-one). After 10 minutes incubation at room temperature, firefly luciferase was measured using a Top-Count Scintillation Counter/Luminescence Reader (Packard, Inc.). Next, 30 μl Stop-Glo substrate (Promega) was added to the cell lysates containing Dual-Glo. After 10 minutes room temperature incubation, *Renilla *luciferase was measured using the same Top-Count Scintillation Counter/Luminescence Reader (Packard, Inc.) For normalization, the firefly luciferase signal was divided by the *Renilla *luciferase signal for the same well. In an experiment, each treatment was applied to a total of four wells.

### RNA Extraction and Real-time, Quantitative PCR

In triplicate, cells stably expressing control or Ubiquilin 1 specific shRNAs were washed twice with cold PBS and total RNA was isolated using the RNeasy Mini Kit (Qiagen, Inc). cDNA was synthesized using total RNA (3.5 μg), N6 random primers (12.5 μM) and SuperScript II Reverse Transcriptase (Invitrogen). cDNAs were diluted 1:30 using RNase-free H_2_O to a final concentration of 2 ng. Diluted cDNAs were mixed with *APP *or *18S *primer/probe sets (Applied Biosystems, Inc.; APP Catalog # Hs00169098_m1; 18S Catalog # Hs99999901_s1), 2× PCR Universal Master Mix (Applied Biosystems, Inc.) and amplified using an ABI 7500 Real-Time PCR System following the manufacturer's directions. To determine differences in *APP *mRNA levels, we utilized the ΔΔCt method.

### Statistical analysis

Values in the text and figures are presented as means ± standard errors of at least three independent experiments. Equal variance or separate variance two-sample student's t-test were used, as appropriate, to compare two groups. Bonferroni correction analysis was used to correct for multiple comparisons within a single experiment.

## Competing interests

RET reports being a consultant or serving on the scientific advisory board and board of directors of Torrey Pines Therapuetics and Prana Biotechnology; holding equity or stock options with Torrey Pines Therapuetics, Prana Biotechnology, and Elan; and having received consulting or lecture fees from Novartis, Aventis Pharma, Eisai, and PureTech Ventures. No other authors reported any potential conflicts of interest. AJS is a shareholder in Torrey Pines Therapuetics.

## Authors' contributions

CZ and PJK designed and carried out the majority of the experiments, data interpretation, and helped draft the manuscript. RC performed QPCR experiments. TLC created the SH-SY5Y-APP-Gal4 stable cell line, performed initial characterization of the AICD-Gal4 luciferase assay. SS established the AICD-Gal4 assay in 96 well format using Sybr green cell number normalization. SAP helped in the characterization of Ubiquilin 1. CPC performed the initial characterization of the AICD-Gal4 luciferase assay. MOC performed statistical analyses. JCL was involved in the statistical analyses, interpretation of results and preparation of the manuscript. RET was involved in the design of the genetic screen and interpretation of results. AJS designed the genetic screen, coordinated the studies, interpreted the results, and drafted the manuscript. All authors have read and approved the final manuscript.
